# Consolidation of a genomic epidemiological surveillance network for tuberculosis (REVIGET) in northern and northeastern Brazil: a study protocol

**DOI:** 10.3389/fpubh.2025.1668926

**Published:** 2025-10-15

**Authors:** Emilyn Costa Conceição, Karla Valéria Batista Lima, Cristiane Cunha Frota, Theolis Costa Barbosa Bessa, Adriana Ayden Ferreira, Abhinav Sharma, Davi Josué Marcon, Layana Rufino Ribeiro, Alex Brito Souza, Danna Karen Corrêa dos Santos, Carlos Augusto Abreu Alberio, Ricardo José de Paula Souza e Guimarães, Ismari Perini Furlaneto, Lilian Eduarda de Oliveira Freitas, Emmily Oliveira Amador, Hendor Neves Ribeiro de Jesus, Leonardo Bruno Paz Ferreira Barreto, Maria Cristina Silva Lourenço, Tulio de Oliveira, Robin Mark Warren, REVIGET Consortium

**Affiliations:** ^1^South African Medical Research Council Centre for Tuberculosis Research, Division of Molecular Biology and Human Genetics, Faculty of Medicine and Health Sciences, Stellenbosch University, Cape Town, South Africa; ^2^Centre for Epidemic Response and Innovation, School of Data Science and Computational Thinking, Stellenbosch University, Stellenbosch, South Africa; ^3^Rede Brasileira de Pesquisas em Tuberculose, Rio de Janeiro, Brazil; ^4^Secao de Bacteriologia do Instituto Evandro Chagas, Ananindeua, Brazil; ^5^Programa de Pos-graduacao em Biologia Parasitária na Amazonia, Universidade do Estado do Pará, Belem, Brazil; ^6^Programa de Pos-graduacao em Epidemiologia e Vigilancia em Saude, Instituto Evandro Chagas, Ananindeua, Brazil; ^7^Departamento de Patologia, Universidade Federal do Ceara, Fortaleza, Brazil; ^8^Instituto Goncalo Moniz, Fundacao Oswaldo Cruz, Salvador, Bahia, Brazil; ^9^Laboratorio Central do Amazonas, Fundacao de Vigilancia em Saude do Amazonas Dra Rosemary Costa Pinto, Manaus, Amazonas, Brazil; ^10^Hospital Universitário Joao de Barros Barreto da Universidade Federal do Para, Belem, Brazil; ^11^Geoprocessamento, Instituto Evandro Chagas, Ananindeua, Brazil; ^12^Centro Universitario do Para, Belem, Brazil; ^13^Laboratorio de Bacteriologia e Bioensaios em Micobacterias, Instituto Nacional de Infectologia Evandro Chagas, Fundacao Oswaldo Cruz, Manguinhos, Rio de Janeiro, Brazil; ^14^Programa de Pos-graduacao de Pesquisa Clinica em Doenças Infecciosas, Instituto Nacional de Infectologia Evandro Chagas, Fundacao Oswaldo Cruz, Manguinhos, Rio de Janeiro, Brazil

**Keywords:** drug-resistant tuberculosis, genomic surveillance, whole-genome sequencing, capacity building, Brazil

## Abstract

Globally, tuberculosis (TB) remains a top cause of death from infectious diseases, with an estimated 1.5 million deaths annually. Given its substantial social and economic burden, TB is a priority in the United Nations 2030 Agenda for Sustainable Development. The WHO’s End TB Strategy emphasizes research, innovation, and the rapid implementation of new technologies, such as whole-genome sequencing (WGS), which are pivotal for precision health approaches and drug-resistant TB (DR-TB) surveillance. This study aims to strengthen genomic TB surveillance in the North and Northeast Brazilian regions by applying WGS to study DR-TB cases, training professionals in genomics and bioinformatics, and deploying a national surveillance platform (GEMIBRA). This is an observational, cross-sectional, prospective, quantitative, and qualitative study to be conducted in the states of Pará, Amazonas, Ceará, and Bahia. A total of 500 *Mycobacterium tuberculosis* complex (MTBC) isolates from DR-TB cases will undergo whole-genome sequencing (WGS), representing 87% of the expected DR-TB cases, including non-tuberculous mycobacteria (NTM) isolates among the DR samples. Data will be analyzed for genotype–phenotype correlations, mutation patterns, and associations with clinical and epidemiological characteristics. Capacity-building activities, including theoretical and hands-on bioinformatics training, will be carried out. The GEMIBRA platform will support data visualization, spatial distribution, and clinical decision-making. The project will generate evidence to validate the contribution of WGS integration in Brazil’s TB surveillance system, support precision treatment approaches, and inform public health interventions. It will also contribute to workforce development and the introduction of decentralized WGS-based diagnostics in resource-limited regions. The project adopts a translational research model and a networked, decentralized approach, facilitating the prompt integration of the knowledge generated into public health practice. Ultimately, this work will contribute to combating TB transmission by identifying transmission dynamics, emerging resistant strains, and informing the National Plan to End TB as a public health problem.

## 1 Introduction

Tuberculosis (TB) remains a curable infectious disease that continues to cause approximately 1.5 million deaths annually worldwide. Due to its profound economic and social burden, TB is prioritized in the United Nations 2030 Agenda for Sustainable Development (1) and remains a central focus for the World Health Organization (WHO) and Brazil’s Ministry of Health. The WHO’s End TB Strategy emphasizes intensified research, innovation, and rapid adoption of advanced technologies—among them, next-generation sequencing (NGS)—to improve TB control and surveillance (2, 3).

Adding complexity to the diagnostic landscape, non-tuberculous mycobacteria (NTM) infections, which can present clinical symptoms similar to TB but require different treatment, are increasingly being recognized as a public health concern. Correct identification of the *Mycobacterium tuberculosis* complex (MTBC; group of bacteria that causes TB) and NTM species is essential for ensuring appropriate therapy, as treatment is highly species-specific (4–6).

In this context, NGS offers several applications in infectious diseases, the most prominent being whole-genome sequencing (WGS), which provides comprehensive coverage of the bacterial genome and is suitable for transmission studies or discovery of novel mutations. In addition, targeted NGS (tNGS), such as Deeplex® Myc-TB (GenoScreen), offers high-coverage sequencing of key particular genes, including drug-resistance-associated genes, and is particularly interesting for detecting heteroresistance and polyclonal infections, as it can be used directly in clinical samples (7, 8).

WGS, however, remains the preferred method for high-resolution genomic surveillance. Unlike the tNGS assays, which are limited to predefined resistance-associated loci, WGS enables the identification of both known and novel mutations across the entire genome, supports phylogenetic analysis for transmission mapping, and allows direct comparison with global genomic datasets (7, 8).

As observed during the COVID-19 pandemic, integrating genomic technologies into public health frameworks is essential. For TB, a One Health approach is increasingly relevant, acknowledging the interplay between socio-environmental factors, host-pathogen interactions, including animals, antimicrobial resistance, and technological advancements (9, 10).

Despite the introduction of new anti-TB drugs, bedaquiline, delamanid, linezolid, and pretomanid, limited knowledge exists regarding their resistance mechanisms. The WHO and the scientific community advocate for expanded genomic surveillance and genotype–phenotype correlation studies. However, such strategies must be adapted to resource-constrained settings, where access to technical infrastructure and trained personnel is limited (11). In the context of NTM, further research is needed to establish a comprehensive catalog of resistance-associated mutations to inform treatment decisions, similar to the resources already available for *Mycobacterium tuberculosis*, the human-adapted MTBC species (12, 13).

Brazil remains among the countries with the highest TB burden globally, belonging to the WHO list of high-burden TB countries (HBC) (3), and a rising clinical relevance of NTM in TB-endemic regions in Brazil is observed (14, 15). Regions such as the North and Northeast of Brazil face disproportionate challenges due to economic disparities, limited laboratory capacity, and delayed diagnosis. Studies have shown that delays in detecting drug resistance are associated with worse clinical outcomes, including death. In addition, systemic inequities rooted in poverty, structural racism, and geographic disparities undermine effective TB control in these regions (16).

It is noteworthy that these regions are also underrepresented in molecular epidemiological studies on TB and face significant limitations in infrastructure to support timely genomic diagnosis and surveillance of this disease (17), challenges that are also relevant to NTM (18). These regions are home to traditional communities, exhibit lower TB cure rates, and have limited access to innovative diagnostic technologies (19). Therefore, there is an urgent need for research that not only advances scientific knowledge but also strengthens local health systems through capacity-building initiatives and the development of context-specific diagnostic tools (18).

## 2 Materials and methods

### 2.1 Study aim and objectives

This study aims to apply WGS to determine the drug resistance profile in cases of drug-resistant TB (DR-TB) and TB-NTM coinfection identified by the national diagnostic laboratory network, and to train healthcare professionals in data analysis, interpretation, and clinical decision-making based on internationally validated methodologies. The project will also implement GEMIBRA, an interactive web-based platform developed to support Brazil’s National Health Data Network (RNDS) in modernizing TB and TB/NTM co-infection surveillance.

The study specific objectives are: (1) To implement WGS as a diagnostic tool for resistance profiling using samples submitted through the regular workflow of the Central Laboratoraies (LACENs) and other diagnostic centers; (2) To conduct genomic and epidemiological analyses of TB and NTM infections; (3) To train laboratory professionals in the North and Northeast regions in WGS, bioinformatics, and data analysis applied to Mycobacteria through theoretical-practical courses, both in-presential and virtually; (4) To deploy the GEMIBRA platform^1^ for facilitating TB surveillance and research through WGS data, including lineage/genotype identification, mutation profiling, phenotypic resistance modeling, transmission patterns, and spatial distribution; (5) To engage health professionals and decision-makers in interpreting WGS results as a potential diagnostic support tool aligned with international guidelines, fostering integration with national systems; (6) To develop educational materials (booklets, books, videos, and apps) and promote health engagement activities to increase TB awareness—particularly drug-resistant TB—among the general public, students, health professionals, and TB patients in diverse educational and social spaces; (7) To evaluate the costs associated with diagnostic methods and the turnaround time within the diagnostic workflow.

### 2.2 Study design and setting

**Figure 1:**
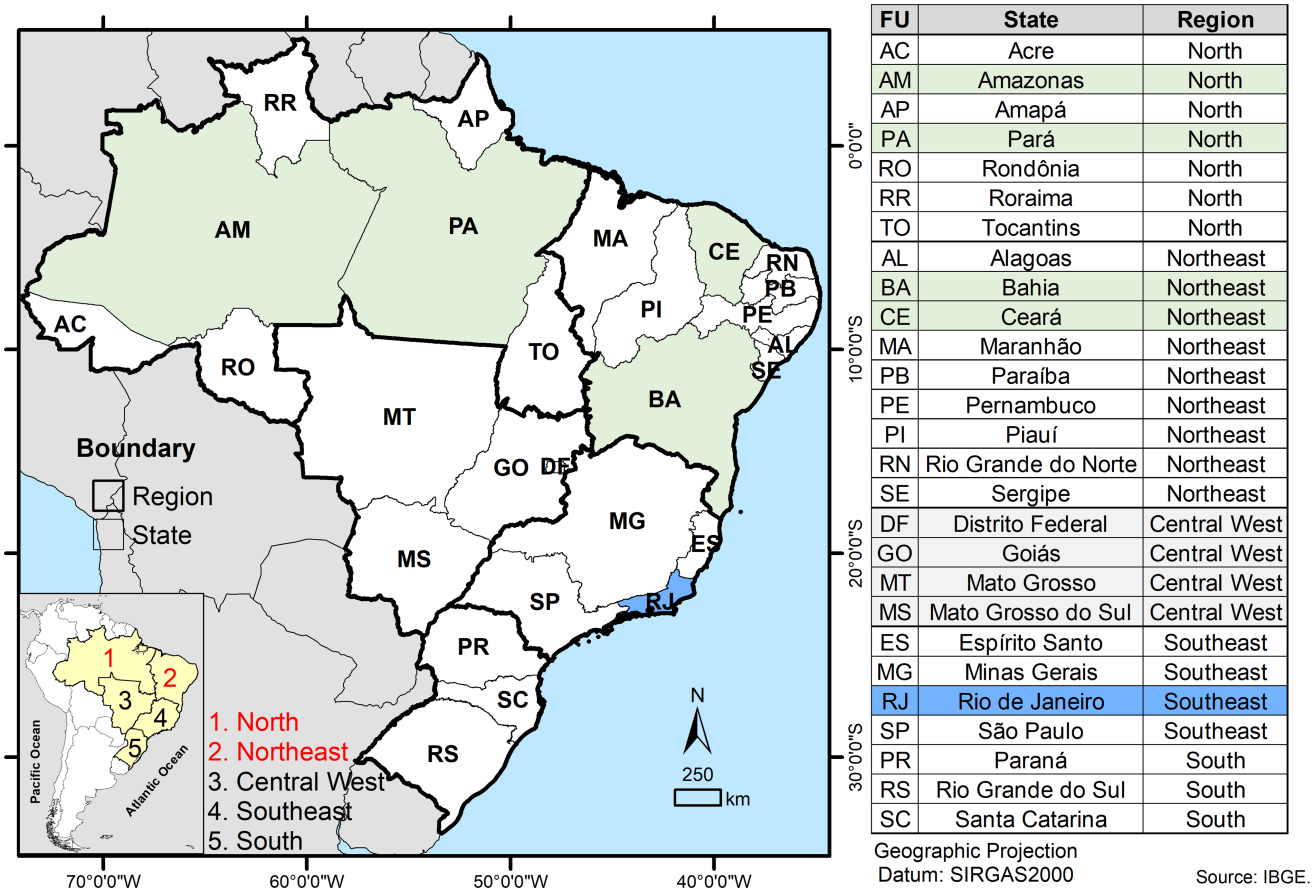
Geographic map of Brazil showing its five major regions and the 27 federal units (states). Study sites involved in sample and data collection, routine diagnostic processing, and capacity-building activities are highlighted in green as part of the North and Northeast regions. The site designated for minimum inhibitory concentration (MIC) testing is indicated in blue.

This is an observational, cross-sectional, prospective, epidemiological, mixed-methods (quantitative-qualitative), descriptive, and analytical study. The study will be conducted for 24 months (2024–2026) in selected locations in the North and Northeast of Brazil, including Ananindeua and Belém (Pará), Manaus (Amazonas), Fortaleza (Ceará), and Salvador (Bahia). The city of Rio de Janeiro (Rio de Janeiro), located in Southeast Brazil, will serve as a reference laboratory to perform the drug susceptibility testing (DST) by determining the minimum inhibitory concentrations (MICs). The study settings are highlighted in Figure 1.

### 2.3 Sample size and eligibility criteria

The study will apply a two-stage sampling approach. In the first stage, a convenience sample will be obtained from all DR-TB cases diagnosed by participating LACENs that meet eligibility criteria (i.e., confirmed culture-positive cases with viable isolates). In the second stage, from within this pool, isolates will be selected through random sampling to reach the target of 500 genomes (approximately 87% of the projected total), ensuring representativeness across states and case categories. Priority will be given to complex cases, including patients with persistent treatment failure, suspected polyclonal infections, or discordant DST results.

Sampling will follow the routine diagnostic workflow and will include *M. tuberculosis* isolates from individuals diagnosed with pulmonary or extrapulmonary DR-TB, confirmed via either solid or liquid culture media. If NTM are later isolated from a sample initially identified as *M. tuberculosis*, whether by Rapid Molecular Test (RMT) or culture, the case will be further assessed to investigate potential TB/NTM coinfection or diagnostic misclassification.

Sample size estimation was informed by internal data provided by each participating LACEN. In 2023, the LACENs of Amazonas, Bahia, Ceará, and Pará reported 176, 152, 125, and 120 cases of DR-TB, respectively, totalling 573 DR-TB cases. Inclusion criteria comprise patients of any age, sex, or nationality diagnosed with any form of DR-TB, regardless of NTM and others co-infections or comorbidities, provided that they are registered in one of the four study states (Amazonas, Bahia, Ceará, or Pará) and have stored samples with viable mycobacterial cultures. Exclusion criteria are being infected with drug-susceptible *M. tuberculosis* or for whom no culture isolates are available.

### 2.4 Sample collection and processing

Sample collection will take place after routine diagnostic testing has been completed at the time when bacterial samples are archived. The recruitment period will be from 01/01/2024 to 31/12/2025. If fewer than 500 DR-TB cases are enrolled across the study sites in 2024, collection will continue into 2025, with priority given to complex DR-TB cases, such as patients experiencing persistent treatment failure, suspected polyclonal infection, or discordant DST results.

The sample collection strategy accounts for the variability in routine TB diagnostic practices across different regions and states within Brazil. In general, people exhibiting clinical symptoms indicative of TB are initially referred to a primary healthcare facility, where sputum specimens are collected for diagnostic evaluation. These specimens are analyzed via microscopy, Ziehl-Neelsen (ZN) staining, and, when available, the RMT GeneXpert MTB/RIF Ultra assay.

Mycobacterial culture is performed on Ogawa medium at the same facility for all samples. Upon confirmation of culture positivity, isolates are forwarded to the LACEN, where each isolate is subcultured on Löwenstein-Jensen (LJ) medium. Once the culture is positive, DST is performed on the first-line drug,s and samples are cryopreserved at −20 °C or −70 °C for traceability. When resistance to rifampicin or isoniazid is detected, then the samples are subcultured in the MGIT instrument for second-line DST and/or the RMT LPA for first and/or second-line drugs when available at the LACEN level; otherwise, they are shipped to their specific regional reference laboratory (RRL). If the test is not performed at the RRL, then it is shipped to the national reference laboratory (NRL) for further second-line DST testing.

**Figure 2:**
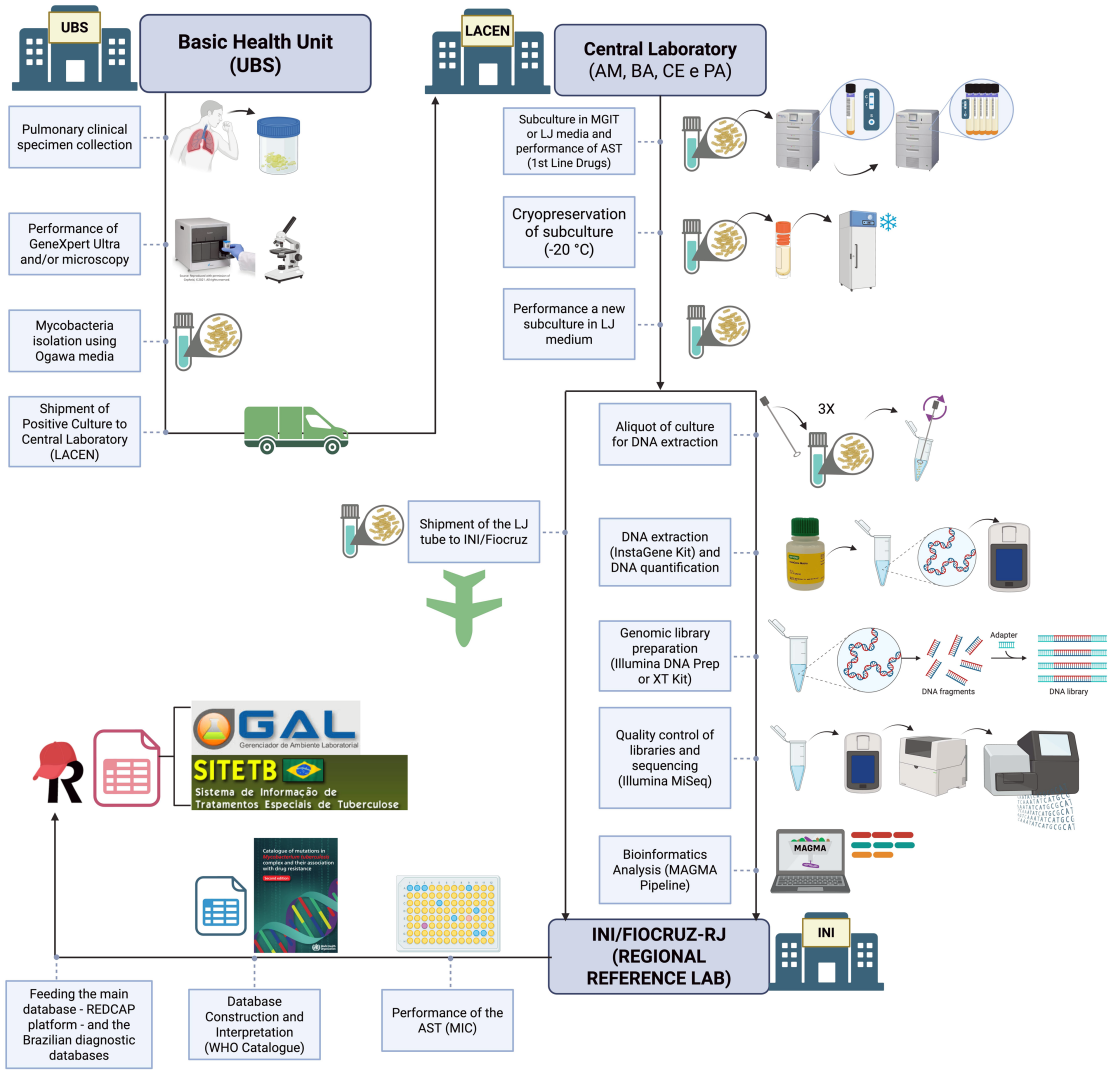
Overview of the protocol workflow from sample collection to data analysis as implemented in the genomic epidemiological surveillance network for tuberculosis (REVIGET) study in Northern and Northeastern Brazil. DST, Drug Susceptibility Test; Basic Health Unit (UBS from the Portuguese name “*Unidade Básica de Saúde*”), LACEN, Central Laboratory from the states of Amazonas (AM), Bahia (BA), Ceará (CE) and Pará (PA); INI, Institute National of Infectiology; LJ, Löwenstein–Jensen; MIC, Minimum Inhibitory Concentration; MGIT, Mycobacteria Growth Indicator Tube. Created in BioRender. Warren, R. (2025), https://BioRender.com/dazlxyj.

This study will be conducted in this scenario as described in Figure 2. The WGS method will be integrated into the routine diagnostic workflow for samples showing resistance to first-line anti-TB drugs and for NTM detected in specimens initially suspected to be TB.

From the routine cryopreserved LJ culture, the Mycobacterial isolate will be subcultured on LJ tubes. An aliquot will be designated for DNA extraction and WGS, while another aliquot will be designated to perform the pDST minimum inhibitory concentration (MIC) method for the following drugs: rifampicin, isoniazid, streptomycin, ethambutol, amikacin, levofloxacin, moxifloxacin, clofazimine, bedaquiline, linezolid, delamanid and pretomanid. The MIC interpretation will follow the Clinical and Laboratory Standards Institute guideline M24 (CLSI) (20). For the MIC test, the samples of the four sites will be shipped to the RRL at the National Institute of Infectology (INI) at Oswaldo Cruz Foundation (FIOCRUZ), in a way not to interfere with the current routine system, and to centralized the phenotypic tests and maintain standardization.

WGS will be performed locally for approximately 36 samples (two rounds on the Miseq Illumina platform). The remaining samples will be sent to the Bacteriology and Mycology Section of the Evandro Chagas Institute, where they will be sequenced on the NextSeq Illumina platform. The LACENs have the Miseq platform, which allows the sequencing of approximately 16 samples at a time, achieving 50-100x coverage. The IEC has both Miseq and NextSeq platforms. Sequencing on the second platform allows the analysis of 70-90 samples with 50-100x coverage, at a reduced cost compared to Miseq. Therefore, due to financial constraints, the remaining samples will be sent to the IEC for sequencing. Between 20 and 30 mycobacterial samples from each LACEN (Amazonas, Bahia, Ceará, and Pará) will be used during practical training sessions focused on capacity building and data analysis. The remaining samples collected throughout the study period will be processed and analyzed by the previously trained team as part of the training follow-up.

For this, sample handling will occur in Biosafety Level 2 plus (BSL-2 +) laboratories. After heat inactivation of cultures at 80 °C for 1 h, samples will be transferred to Biosafety Level 2 (BSL-2) laboratories for downstream procedures. Laboratory personnel will strictly follow locally approved biosafety protocols and standard operating procedures, including the continuous use of personal protective equipment (PPE) and appropriate handling of reagents and specimens.

The mycobacterial DNA will be extracted from solid culture on LJ using the InstaGene (IGM) Matrix kit (Bio-Rad, United States) as per Conceicao et al. protocol with modifications, which includes the use of a Vortex for 5 min instead of a high-speed homogenizer instrument (21). The DNA quantification in ng/uL will be assessed using a Qubit Fluorometer (Thermo Fisher Scientific, Waltham, United States) double-strand High Sensitive (dsHS) kit.

Following, the genomic DNA samples with a minimum of 0.5 ng/uL in a volume of 150 uL IGM Matrix will be selected for library preparation using the DNA Prep kit or Nextera XT kit (Illumina, Santa Clara, United States), depending on their availability. The genomic libraries’ quality control will be assessed using a Qubit Fluorometer dsHS kit for quantification (ng/uL) and TapeStation system for fragment size in base pairs (bp) verification using a D5000 kit whose range is expected to be around 400–600 bp. The sequencing will be performed using Illumina MiSeq platforms, which are already installed at the LACENs.

The Bioinformatics analysis will use the MAGMA pipeline (22), and drug resistance interpretation will follow the WHO catalog of mutations (Second Edition) (12). WGS results and phenotypical profile will be interpreted using WHO’s mutation catalog, and all validated results are subsequently uploaded to the REDCap database and Brazilian diagnostics databases Laboratory Environment Manager (GAL)^2^ and Special Tuberculosis Treatment Information System (SITE-TB).^3^

To guarantee the robustness of WGS implementation, we will adopt a multi-tier quality assurance strategy. Internal quality control will include monitoring DNA extraction yield and purity, library preparation success, and sequencing metrics as previously described (e.g., coverage depth >30×, genome completeness >95%). Control strains (positive controls: H37Rv, MDR reference isolates; and negative control: purified water) will be included in each sequencing run to ensure reproducibility.

External validation will be conducted by periodically re-sequencing a subset of isolates and comparing results. Data analysis will follow WHO-endorsed bioinformatics standards, including the WHO mutation catalog (13), ensuring international comparability. These measures will ensure consistency and reliability during the implementation phase.

### 2.5 Data collection and management plan

Brazil has an established National Network of Databases in Health (RNDS).^4^ This secure system connects health databases nationwide, ensuring privacy under the General Law of Data Protection (LGPD) and the Law of Access to Information (LAI) (20, 23). Among the databases available, the GAL and the SITE-TB are the primary sources of data concerning laboratory, geographical, sociodemographic, epidemiological, and clinical information, including monthly follow-up, medication regimens, and treatment outcomes.

The diagnostic laboratories and reference hospitals from each participating region are responsible for collecting and managing primary data. A de-identified, linked database containing variables from the GAL and SITE-TB subsystems will be provided by each state’s secretary of health for evaluation in connection with the genomic data obtained. This dataset will include individuals diagnosed with DR-TB between January 2024 and December 2025. Data collection will take place from January to December 2025.

Study data will be collected and managed using REDCap electronic data capture tools hosted at Oswaldo Cruz Foundation - Ageu Magalhǎes Institute (Fiocruz/IGM). REDCap (Research Electronic Data Capture) is a secure, web-based software platform designed to support data capture for research studies, providing (1) an intuitive interface for validated data capture; (2) audit trails for tracking data manipulation and export procedures; (3) automated export procedures for seamless data downloads to common statistical packages; and (4) procedures for data integration and interoperability with external sources (21, 22). Upon completion of the analysis, only aggregated analyses and de-identified genomic and phenotypic data of the bacterial strains will be made publicly available.

The project will be coordinated using the Notion platform^5^ for task management. Periodic weekly remote meetings will ensure monitoring of the project’s progress. Budget and administrative risks will be managed with support from the Centre of Excellence in Project Management (*Núcleo de Excelência em Gestão de Projetos*) of the Gonçalo Moniz Institute, Oswaldo Cruz Foundation, Bahia (NEGEP/IGM/Fiocruz-BA).

### 2.6 Capacity building in WGS and bioinformatics

Capacity building is a central component of this collaborative initiative between Brazil and South Africa. Training activities will be coordinated by two representatives from Stellenbosch University, affiliated with the TB Genomics Research Group, the Bioinformatics Unit, and the Center for Epidemic Response and Innovation (CERI), with support from two previously trained personnel from the IEC.

Initial training sessions will be held at the state public health laboratories (LACENs) in Amazonas, Pará, Bahia, and Ceará. Each session will consist of a one-week intensive course (40 h), featuring a public workshop conducted in Portuguese and offered both in person and online.

The training program will cover: (1) theoretical foundations of next-generation and whole genome sequencing, including their applications in public health; (2) hands-on laboratory sessions encompassing DNA extraction, library preparation, quality control, and sequencing procedures; and (3) bioinformatics modules focused on pipeline execution, data analysis, and the generation of patient-centered reports.

Follow-up training sessions will be held monthly, comprising both theoretical and practical components in bioinformatics. These will be supplemented by additional activities and assessments to reinforce learning outcomes and ensure the continuous development of trainees’ competencies and skills.

### 2.7 Development of the GEMIBRA platform

The development of GEMIBRA^6^ will occur in four stages: (1) retrieval, QC, and validation of public MTBC genomes; (2) data analysis using the MAGMA pipeline,^7^ which identifies lineage, genotype, resistance profile, transmission dynamics, and supports clinical decision-making; (3) integration with geospatial data to map transmission by state; and (4) development, validation, and deployment of an interactive user interface for researchers and health professionals.

### 2.8 Statistics and data analysis

Sociodemographic and clinical-epidemiological characteristics will be analyzed through descriptive statistics using frequencies for categorical variables, and mean and standard deviation (SD) for parametric variables with a 95% confidence interval (95% CI), or median and interquartile range (IQR) for non-parametric variables. Normality will be evaluated using Shapiro–Wilk test. A significance level of 5% (*α* = 0.05) will be applied. Statistical analyses will be conducted using GraphPad Prism for MAC (version 10.4.2 or later).

Bioinformatics analysis will be performed using the MAGMA pipeline, which is optimized for analyzing MTBC genomes, including contaminated samples. To assess the cost of diagnostic methods, both Cost-Consequence Analysis (CCA) and Cost-Effectiveness Ratios (CER) will be applied. For the CCA, the annual acquisition cost, diagnostic accuracy, and turnaround time of each test will be compiled in a Microsoft Excel 365 matrix. The CER will be derived by comparing test costs against diagnostic quality and speed, thus informing the annual cost-efficiency of each method.

### 2.9 Ethical considerations

This study was approved by the institutional review board of Instituto Evandro Chagas (CAAE: 85134924.4.0000.0019), in compliance with the Brazilian legislation (Res. CNS n° 466/2012; Brazilian Law 12.527, November 18th, 2011; Brazilian Law 14.874, May 28th, 2024; and their regulations), and the Declaration of Helsinki. It was also registered in the National System for the Management of Genetic Heritage and Associated Traditional Knowledge (SisGen), under the Registration No. A247AD9, in compliance with the Brazilian Law 13.123, May 20th, 2015.

The Institutional Review Board (IRB) waived the requirement for informed consent based on several considerations. First, the study will use retrospective data and bacterial isolates that have already been collected and archived as part of Brazil’s routine TB surveillance and care system. Reaching out to all individuals to obtain consent is impractical due to the large number of cases and would introduce selection bias for surveillance, potentially compromising the scientific validity of analyses aimed at assessing genetic diversity and drug resistance mutations in *M. tuberculosis. S*econd, the information to be analyzed consists exclusively of de-identified data already available in national public health databases (GAL and SITE-TB), routinely collected for compulsory disease notification and treatment monitoring. No additional variables beyond those present in these systems will be requested. The new data to be integrated, such as WGS results and MIC will also be generated by the same public laboratories (LACENs and reference labs) already responsible for the surveillance data, in alignment with their mandate. As a public health surveillance study conducted by institutions under the Ministry of Health (e.g., IEC or Fiocruz), this project operates under the legal framework of Brazil’s Organic Health Law and the Law on Access to Information. In this context, obtaining individual informed consent is not applicable. All data will be fully anonymized prior to analysis. If necessary, limited review of medical records may occur, but only to confirm or complete surveillance-relevant variables already collected in the official systems.

Importantly, the results generated in this project will be communicated back to state TB programs and treating physicians through integration with GAL and SITE-TB. This will allow genomic resistance profiles to directly inform treatment decisions, reducing the time to appropriate therapy and improving clinical outcomes. By providing individualized resistance data, WGS will contribute to precision medicine within the Unified Health System (*Sistema Único de Saúde - SUS*) framework, ensuring that patients benefit from optimized regimens while minimizing the risk of treatment failure or further amplification of resistance.

### 2.10 Safety considerations

This study involves potential risks related to laboratory procedures, administrative processes, data management, and data interpretation. To ensure the safety of participants and personnel, as well as the integrity of the research, multiple preventive and mitigation strategies will be implemented.

Laboratory safety will be a priority throughout the study, and biosafety measures will be followed to ensure a safe working environment for all staff involved, following the established regulatory standard No. 32 “Occupational Safety and Health in Health Care Services” (24). All clinical specimens and *M. tuberculosis* cultures will be processed following established biosafety regulations. The risk level in a TB laboratory refers to the likelihood of laboratory personnel becoming infected with *M. tuberculosis* due to procedures performed in the laboratory.

Activities associated with a low risk involve a minimal likelihood of generating infectious aerosols from clinical specimens and typically involve a low concentration of infectious particles. Such activities include direct smear microscopy, sample preparation for automated nucleic acid amplification tests (e.g., RMT), and the swab method for bacterial isolation (culture in Ogawa-Kudoh). These can be performed outside a Biological Safety Cabinet (BSC) class II, in case they are conducted near a Bunsen burner and with appropriate use of personal protective equipment (PPE) (25, 26).

**Figure 3:**
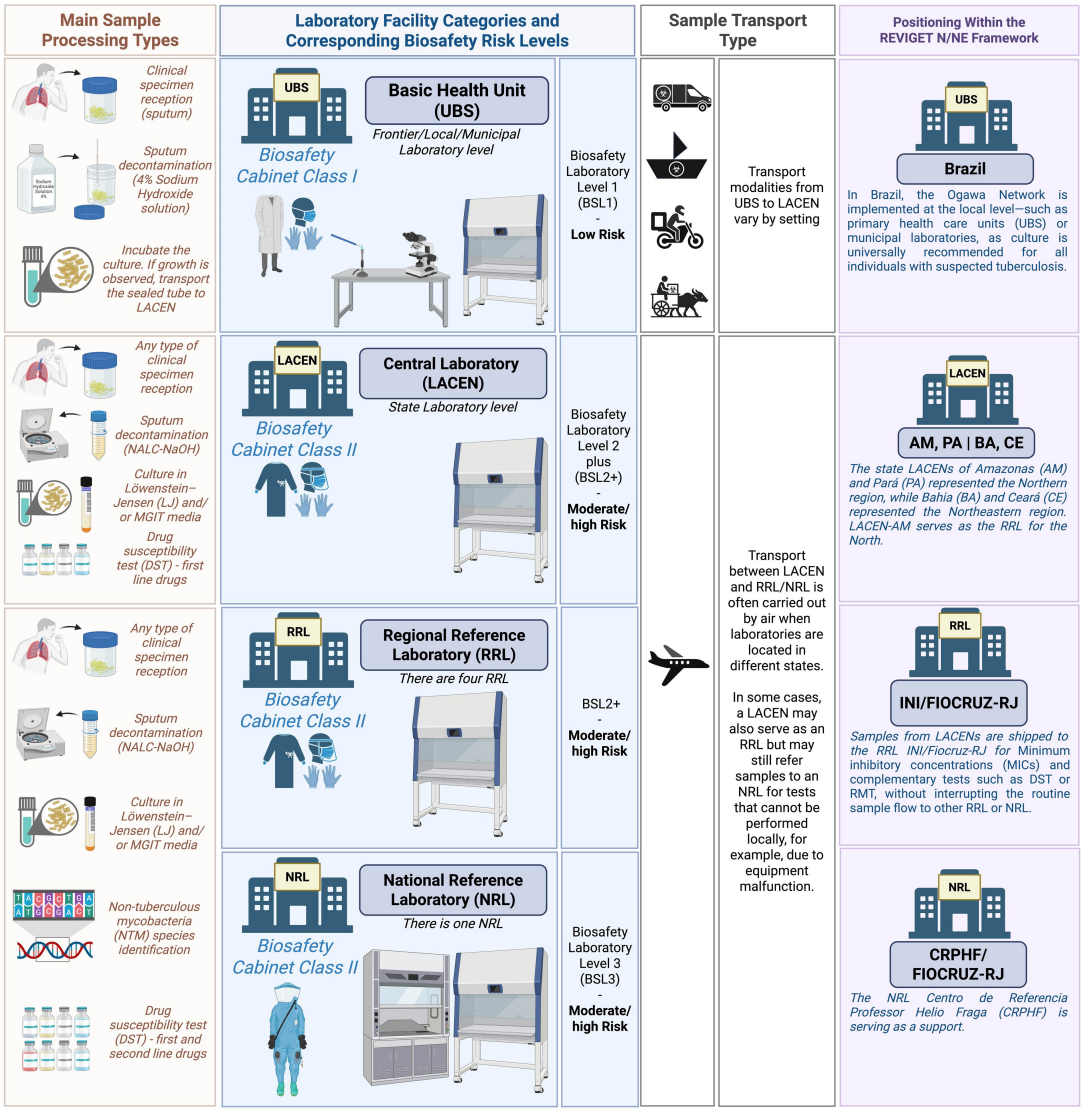
Schematic representation of sample processing complexity, laboratory biosafety requirements, transport logistics, and study integration within the national diagnostic and surveillance framework. Created in BioRender. Warren, R. (2025) https://BioRender.com/dazlxyj.

Procedures associated with a moderate risk involve a moderate potential for aerosol generation but still involve low concentrations of infectious particles. These include processing and concentration of biological samples for inoculation into primary isolation media (e.g., Löwenstein-Jensen or MGIT) and direct drug susceptibility testing, such as the Line Probe Assay (LPA), on processed sputum samples using centrifuge equipment, for example. These activities must be performed within a BSC and require proper use of PPE. High-risk activities are those with a high potential for generating infectious aerosols and involve high concentrations of infectious particles. These include manipulation of cultures grown in solid or liquid media for species identification, phenotypic drug susceptibility testing, or LPA. All such procedures must be conducted within a BSC with strict adherence to PPE protocols (Figure 3) (26).

In Brazil, a national program known as the Ogawa-Kudoh Network promotes universal culture for individuals with suspected tuberculosis. The Ogawa-Kudoh method, also referred to as the K-O swab method, is widely used for culturing *M. tuberculosis* from sputum samples, particularly in resource-limited settings.

A critical step in this technique is sputum decontamination, which eliminates contaminating bacteria and debris while preserving viable mycobacteria. The procedure involves collecting sputum with a sterile swab, immersing it in a 4% sodium hydroxide solution for decontamination, and subsequently inoculating the swab onto Ogawa medium. Due to its operational simplicity, low cost, and minimal infrastructure requirements, this method is routinely implemented in laboratories at the local/municipal levels, such as the Basic Health Unit (UBS) (26).

The disposal of biohazardous waste in this study will be according to Brazilian Law No. 12.305/2010, known as the National Solid Waste Policy (*Política Nacional de Resíduos Sólidos – PNRS*), Brazilian regulatory norm No. 12809 regarding health service waste manipulation and ANVISA regulatory norm (RDC) No. 222/2018, which specifically addresses the management of healthcare service waste. This legislation establishes guidelines for the integrated management and environmentally sound handling of solid waste, promoting practices such as waste reduction, reuse, recycling, and appropriate final disposal (27–29). In addition, the National Health Surveillance Agency (ANVISA) and the National Council for the Environment (CONAMA) play key roles in overseeing and regulating laboratory waste disposal practices.

Culture transportation will follow the technical guidelines for biological risk, classifying the material as Risk Group B. It will be packaged and labeled with UN 2814 substance identification code, accompanied by the required documentation as specified in ANVISA’s Manual for Transport of Human Biological Material, in compliance with IATA regulations (26, 30).

Administrative risks may include challenges in procurement, budgeting, and travel logistics. These will be addressed through collaboration with a dedicated and trained technical management team responsible for supporting project operations and ensuring smooth coordination across sites.

Risks to the institutional image could arise from procedural errors or infrastructural limitations that impact the execution of laboratory activities. These risks will be minimized through ongoing technical oversight and quality control. The project team has demonstrated experience in delivering high-quality training and has received strong performance evaluations in previous initiatives, which supports confidence in the implementation of this study.

Data management and privacy are also critical considerations. This study will follow the General Data Protection Law No. 13.709/2018 (*Lei Geral de Proteção de Dados Pessoais – LGPD*), which establishes regulations governing the collection, processing, storage, and sharing of personal data in Brazil. Its primary objective is to safeguard fundamental rights related to freedom, privacy, and the free development of the individual.

Mishandling of data could compromise patient confidentiality, and laboratory staff may face exposure to multidrug-resistant strains of *M. tuberculosis*. To mitigate these risks, all patient data will be anonymized, and access to sensitive information will be restricted to the study coordinator and designated team members using a secure REDCap database. This will be important for the identification of serial isolates and further investigation when necessary.

Lastly, the potential for misinterpretation of genomic and epidemiological correlations will be addressed through rigorous data analysis using appropriate statistical models and expert review. All interpretations will be collaboratively evaluated by the multidisciplinary research team to ensure scientific accuracy and alignment with the study objectives.

### 2.11 Project status and timeline

The project activities will be executed from 2024 to 2026. These activities are organized into 10 Work Packages (WPs): WP1: Project Management and Data Infrastructure; WP2: Ethical and Epidemiological Requirements; WP3: Clinical Ecosystem; WP4: Laboratory Ecosystem; WP5: Bioinformatics and Digital Tools; WP6: Data Analysis; WP7: WGS Integration into RNDS and GEMIBRA; WP8: Development of Educational Materials; WP9: Capacity Building, Dissemination, and Scientific Communication; WP10: Political and Social Engagement. The timeline is detailed within the Supplementary Table 1.

### 2.12 Communication of results, health promotion, and community engagement

Strategies for translating and disseminating knowledge about DR-TB diagnosis and treatment will be developed in partnership with civil society organizations, healthcare professionals, and health authorities. The project will promote health education and scientific outreach adapted to diverse audiences through educational activities, publications, and community engagement. Results will be disseminated through peer-reviewed journal articles, academic presentations, and public-facing events. These initiatives will take place concurrently with the laboratory and data management phases of the project, ensuring real-time communication and feedback.

## 3 Discussion

This protocol describes the implementation of the REVIGET network, which aims to integrate WGS into routine DR-TB surveillance in Brazil, with a focus on DR-TB in historically underserved regions. While several countries, including South Africa, India, the United Kingdom, and members of the European Union, have already established national genomic surveillance systems, few initiatives have prioritized decentralized implementation in low-resource settings. Lessons from South Africa and India, for example, highlight both the transformative value of WGS for rapid resistance detection and the challenges of scaling laboratory and bioinformatics capacity in regions with high TB burden (11, 18).

REVIGET builds on these experiences but introduces specific innovations that differentiate it from previous efforts. First, it focuses on the North and Northeast of Brazil, regions with disproportionately high TB incidence and mortality, where diagnostic inequities persist. Second, it integrates TB and NTM surveillance, acknowledging the growing public health impact of NTM in endemic regions. Third, REVIGET is coupled with GEMIBRA, a digital platform aligned with Brazil’s National Health Data Network (RNDS), ensuring that genomic data are rapidly accessible for both clinical decision-making and public health action.

In this sense, REVIGET contributes to the global genomic surveillance network by expanding coverage to a geographic area underrepresented in international datasets and by providing a model for implementing WGS in resource-constrained contexts. By aligning with WHO recommendations and establishing interoperability with international standards, the project ensures that Brazilian data can contribute directly to global resistance monitoring efforts. Moreover, by emphasizing capacity building and equity, REVIGET demonstrates how genomic surveillance can be used as a tool not only for scientific discovery but also for health system strengthening and social justice in TB control.

REVIGET’s implementation represents an important process innovation, incorporating WGS into DR-TB surveillance workflows. This has the potential to enhance case detection, accelerate treatment decisions, and support precision medicine by generating individualized resistance profiles and tracking transmission dynamics. Identifying novel mutations is highly important; however, understanding the mutations present in circulating strains is equally crucial, particularly for evaluating the sensitivity and specificity of new molecular methods in our setting (31). Moreover, integration with the National Health Data Network (RNDS) via the GEMIBRA platform will contribute to more timely, actionable insights across TB care continuously.

Scientific and technical outputs expected from this project include new insights into the genomic diversity and evolutionary pathways of MTBC, a better understanding of resistance mechanisms to newly introduced drugs (e.g., bedaquiline, delamanid, linezolid and pretomanid), and the generation of high-resolution epidemiological data in geographically historically underrepresented areas in TB genomics research.

Our findings will be disseminated through peer-reviewed publications, technical manuals, scientific symposia and training events. These are crutial aspects which should equally take priority in translational research. As Brownson et al. demonstrated, each research setting has unique characteristics, and a “one size fits all” approach is unlikely to succeed in building diversity and inclusion capacity. However, many of the strategies and lessons learned can be adapted, replicated, and refined to fit local contexts (32). The project also anticipates the formation of master’s and PhD students, contributing to long-term workforce development in genomics, surveillance, and bioinformatics.

Dissemination strategies will extend beyond scientific audiences to reach healthcare workers, patients, and the public. Materials such as printed guides, videos, and a mobile application (“TudoTB”) will promote health literacy and patient empowerment, particularly among vulnerable populations. Community engagement activities will involve civil society organizations, local TB committees, and public health authorities in participating states. These initiatives will address TB/HIV coinfection, treatment adherence, stigma situation, and healthcare access in historically underserved populations, including quilombola, riverside, Indigenous, and people deprived of liberty communities. These activities are crucial for sustainability purpose, as community engagement enhances the implementation of evidence-based interventions across diverse settings, increasing their effectiveness in underserved populations and advancing health equity (33). This project also promotes equity by ensuring representation and capacity-building in underserved regions, fostering inclusive research environments. Participatory approaches, such as local training, stakeholder meetings, and collaboration with advocacy committees, will guide the project’s implementation and help shape locally appropriate responses to DR-TB.

A potential limitation of this study is its reliance on culture-based workflows for WGS, which may lead to the exclusion of samples with delayed or failed growth, particularly in complex or extrapulmonary TB cases. This limitation could introduce bias in strain representation, potentially underestimating genomic diversity or specific resistance mutations. Moreover, implementation may face logistical constraints, including reagent shortages or delays in procurement and training across multiple regions. These risks will be addressed through contingency planning, centralized oversight and adaptive scheduling.

Preliminary results from the Brazilian Ministry of Health based on 2024 data demonstrated that between 2015 and 2024, a total of 20,628 new cases of DR-TB were reported in Brazil, including 1,047 cases registered in 2024. The historical trend reveals fluctuations over the years, with notable declines in 2016 and 2020, likely associated with stock shortages of the RMT Xpert MTB/RIF cartridges and the impact of the COVID-19 pandemic, respectively. From 2021 onward, a gradual recovery in case notifications was observed, peaking in 2022 with 1,231 reported cases, followed by a reduction in 2024 (*n* = 1,047). The implementation of the RMT Network for TB in 2014 might have played a key role in this scenario, contributing significantly to the increased detection of RR-TB (34).

It is important to highlight that the spatial distribution analysis of newly diagnosed DR-TB cases between 2016 and 2024 indicates that all federal units (FUs) reported at least one case during this period. From 2016 to 2019, 3,875 new DR-TB cases were notified, with higher concentrations in the municipalities of Rio de Janeiro (*n* = 547; 14.1%), Manaus (*n* = 202; 5.2%), Porto Alegre (*n* = 202; 5.2%), São Paulo (*n* = 192; 5.0%), and Belém (*n* = 156; 4.0%). These same cities also recorded the highest number of DR-TB cases from 2020 to 2024, accounting for a combined total of 5,170 new cases: Rio de Janeiro (*n* = 651; 12.6%), Manaus (*n* = 397; 7.7%), São Paulo (*n* = 394; 7.6%), Belém (*n* = 245; 4.7%), and Porto Alegre (*n* = 176; 3.4%). Despite the negative impact of the COVID-19 pandemic on TB detection and follow-up, the number of DR-TB notifications increased by 33.4% from 2020 to 2024 compared to the period from 2016 to 2019 (34).

In 2023, the highest TB mortality rates in the country were recorded in the states of Amazonas (5.1 deaths per 100,000 population), Pernambuco (4.8 deaths per 100,000), and Rio de Janeiro (4.6 deaths per 100,000). Furthermore, 12 federal units reported mortality rates above the national average of 2.85 deaths per 100,000 population. The North region stood out with four states exhibiting mortality rates above 3.5 per 100,000, highlighting significant regional disparities in the response to the disease (34).

In 2024, TB incidence rates varied across the federal units as in previous years. The states with the highest incidence rates were Amazonas (94.7 cases per 100,000 population), Rio de Janeiro (75.3 cases per 100,000), and Roraima (64.3 cases per 100,000). Additionally, the North region concentrated five states with incidence rates above 50 cases per 100,000 population: Amazonas, Roraima, Pará (61.8), Acre (58.3), and Amapá (52.9), reinforcing the persistent regional inequalities in TB burden (34), which endorses initiatives as REVIGET N/NE, directed to attend the different regional aspects of TB dynamic within the complexity of a large and diverse country as Brazil.

Any protocol amendments, including modifications to sampling strategy, timelines, or analytical workflows, will be documented and submitted for ethical review through the Brazilian “*Plataforma Brasil”* system.^8^ If unforeseen challenges arise that compromise data integrity, participant safety, or the feasibility of project activities, study termination will be considered through consultation with institutional partners, funders, and ethics committees. Transparent reporting and documentation of any changes will be maintained throughout.

## Footnotes

^1^ http://www.ioc.fiocruz.br/gemibra/

^2^ http://gal.datasus.gov.br/GALL/index.php?area=01

^3^ http://sitetb.saude.gov.br/

^4^ https://www.gov.br/saude/pt-br/composicao/seidigi/rnds

^5^ https://www.notion.so/

^6^ http://www.ioc.fiocruz.br/gemibra/

^7^ https://github.com/TORCH-Consortium/MAGMA

^8^ https://plataformabrasil.saude.gov.br/login.jsf

## The group members of REVIGET consortium

**Brazil, Pará team (26 members)**: Alex Brito Souza^1,2^, Allana Aymê Farias Batalha^3^, Ana Judith Pires Garcia Quaresma^1^, Ana Paula Sousa Araújo^3^, Carlos Augusto Abreu Alberio^4^, Danna Karen Corrêa dos Santos^1,2^, Davi Josué Marcon^1,2^, Emilly Gabriele Ribeiro Dias^1,2^, Emmily Oliveira Amador^1,2^, Erilene Cristina da Silva Furtado^3^, Gleissy Adriane Lima Borges^3^, Ismari Perini Furlaneto^6^, José Anderson Monteiro Pantoja^1^, Juan Pereira Soares^1^, Karla Valéria Batista Lima^1,2,5^, Katia Cristina de Lima Furtado^3^, Layana Rufino Ribeiro^1,5^, Lúcia Helena Martins Tavares Monteiro^7^, Luna Luana de Jesus Pantoja^3^, Maria Isabel Montoril Gouveia^1,5^, Patricia Miriam Sayuri Sato Barros da Costa^3^, Rafaella Bonfim Barros^3^, Rosa Márcia Saraiva Gentil^3^, Ricardo José de Paula Souza e Guimarães^8^, Valnete das Graças Dantas Andrade^3^, Victor Cezar Gomes Melo^3^**. Brazil, Amazonas team (13 members)**: Adriana Ayden Ferreira^9^, Claudio Fernández Araujo^9^, Etelvina das Graças da Costa Zaranza^9^, Gustavo Kiyoshi Massunari^9^, Katiuscia Dantas de Araújo^9^, Layssa do Carmo Barroso^9^, Lilian Eduarda de Oliveira Freitas^9^, Marco Aurélio Almeida de Oliveira^9^, Marlucia da Silva Garrido^9^, Reinaldina Dorotheia Nascimento Vieira^9^, Renata Lia Coragem Carvalho^9^, Tatyana Costa Amorim Ramos^9^, Walter André Junior^9^. **Brazil, Bahia team (11 members)**: Andrezza Kariny Miranda de Souza^10^, Erivelton Oliveira^11^, Felicidade Mota Pereira^11^, Gabriela Sant’Ana Menezes de Andrade^11^, Hendor Neves Ribeiro de Jesus^10^, Ingrid Pimentel Silva Gabriel^10,11^, Marcela Kelly Astete Gomez^12^, Rodrigo Alves da Costa Goncalves^10^, Theolis Costa Barbosa Bessa^10,13^, Tonya Azevedo Duarte^10,14^, Vanessa Brandão Nardy^12^. **Brazil, Ceará team (13 members)**: Alex Pessoa da Rocha^15^, Ana Karoliny Alves^16^, Antonia Cely Vitor Barbosa^15^, Cristiane Cunha Frota^16^, Karene Ferreira Cavalcante^15^, Larissa Leão Ferrer de Sousa^15^, Michelle Guilherme de Lima^15^, Rosiane Marcelino Lobo Fernandes^15^, Shirlene Telmos Silva de Lima^15^, Sidarta Lopes Viana^15^, Soraya de Oliveira Sancho^16^, Thiago Pinto Brasil^16^, Vânia Angelica Feitosa Viana^15^. **Brazil, Rio de Janeiro team (6 members):** Leonardo Bruno Paz Ferreira Barreto^17, 18^, Maria Cristina da Silva Lourenço^18^, Yrneh Prado Palacios^18^, Philip Noel Suffys^19^, Fátima Fandinho^20^, William Marco Vicente da Silva^20^. **South Africa, Stellenbosch University team (4 members):** Abhinav Sharma^21^, Emilyn Costa Conceição^13,21,22^, Robin Mark Warren^21^, Tulio de Oliveira^22^. Affiliations: 1. Seção de Bacteriologia, Instituto Evandro Chagas, Ananindeua, Pará, Brazil. 2. Programa de Pós-Graduação em Biologia Parasitária na Amazônia, Universidade do Estado do Pará, Belém, Brazil. 3. Laboratório Central do Estado do Pará, Belém, Pará, Brazil. 4. Hospital Universitário João de Barros Barreto, Ambulatório de Tuberculose Multirresistente, Universidade Federal do Pará, Belém, Pará, Brazil. 5. Programa de Pós-graduação em Epidemiologia e Vigilância em Saúde, Instituto Evandro Chagas, Ananindeua, Brazil. 6.Centro Universitário do Pará, Belém, Pará, Brazil. 7. Secretaria de Estado da Saúde do Pará, Belém, Pará, Brazil. 8. Seção de Geoprocessamento, Instituto Evandro Chagas, Ananindeua, Pará, Brazil. 9. Laboratório Central do Amazonas, Fundação de Vigilância em Saúde do Amazonas Dra Rosemary Costa Pinto, Manaus, Amazonas, Brazil. 10. Fundação Oswaldo Cruz, Instituto Gonçalo Moniz, Salvador, Bahia, Brazil. 11. Programa de Pós-graduação em Biotecnologia em Saúde e Medicina Investigativa, Fundação Oswaldo Cruz, Salvador, Bahia, Brazil. 12. Laboratório Central do Estado da Bahia, Salvador, Bahia, Brazil. 13. Rede Brasileira de Pesquisas em Tuberculose, Rio de Janeiro, Rio de Janeiro, Brazil. 14. Instituto de Ciências da Saúde, Universidade Federal da Bahia, Salvador, Bahia, Brazil. 15. Laboratório Central do Estado do Ceará, Fortaleza, Ceará, Brazil. 16. Departamento de Patologia e Medicina Legal, Faculdade de Medicina, Universidade Federal do Ceará, Fortaleza, Ceará, Brazil. 17. Laboratório de Bacteriologia e Bioensaios em Micobactérias, Instituto Nacional de Infectologia Evandro Chagas, Fundação Oswaldo Cruz, Manguinhos, Rio de Janeiro, Rio de Janeiro, Brazil. 18. Programa de Pós-Graduação em Pesquisa Clínica e Doenças Infecciosas, Instituto Nacional de Infectologia Evandro Chagas, Fundação Oswaldo Cruz, Manguinhos, Rio de Janeiro, Rio de Janeiro, Brazil. 19. Laboratório de Biologia Molecular Aplicada a Micobactérias, Instituto Oswaldo Cruz, Fundação Oswaldo Cruz, Rio de Janeiro, Brazil. 20. Laboratório de Referência Nacional de Tuberculose e Micobacterioses Angela Maria Werneck, Centro de Referência Professor Hélio Fraga, Escola Nacional de Saúde Pública, Fundação Oswaldo Cruz, Rio de Janeiro, Brazil. 21. South African Medical Research Council Center for Tuberculosis Research, Division of Molecular Biology and Human Genetics, Faculty of Medicine and Health Sciences, Stellenbosch University, Cape Town, South Africa. 22. Center for Epidemic Response and Innovation, School of Data Science and Computational Thinking, Stellenbosch University, Stellenbosch, South Africa.
