# Supplementary material for: Consolidation of a genomic epidemiological surveillance network for tuberculosis (REVIGET) in northern and northeastern Brazil: a study protocol
Source: Front Public Health. 2025 Oct 15;13:1668926. doi: 10.3389/fpubh.2025.1668926 (PMC12568509; doi:10.3389/fpubh.2025.1668926)
Supplement: Supplementary file 1 [file Table_1.DOCX]

**Supplementary Table 1:** Phases of the Study and Planned Timeline of Activities Over 24 Months.

| **Month** | **Phase 1: Strategic Planning** |
| --- | --- |
| 1-3 | Stakeholder engagement and strategic alignment meetings |
|  | Preparation of standard operating procedures (SOPs) |
|  | Submission of protocol to relevant Research Ethics Committees |
|  | Finalization of logistics and training agendas for WPs 1 to 10 |
| 4-20 | **Phase 2: Training and SOP Implementation** |
|  | Development and validation of SOPs and training manuals |
|  | Delivery of theoretical and practical training workshops |
|  | Remote training and supervision through virtual platforms |
| 4-22 | **Phase 3: Sample Collection and Data Acquisition** |
|  | Inclusion of retrospective and prospective samples collected from 2024 onwards |
|  | Clinical and laboratory data acquisition from participating sites |
| 6-22 | **Phase 4: Whole Genome Sequencing (WGS) and Bioinformatic Analysis** |
|  | Execution of WGS and downstream analysis using MAGMA and other tools |
|  | Quality control and integration of findings with phenotypic and clinical data |
| 7-22 | **Phase 5: Monitoring of DR-TB Cases and Decentralized Activities** |
|  | Use of WGS data to support clinical management of complex DR-TB cases |
|  | Definition of complex cases includes suspected polyclonal infections, resistance progression, or therapeutic failure |
|  | Sample sharing between laboratories for quality assurance and result validation |
| 10-24 | **Phase 6: Scientific Output and Communication** |
|  | Presentation of partial and final results at regional, national, and international scientific events |
|  | Submission of manuscripts for peer-reviewed publication |
| 3-24 | **Phase 7: Health Education** |
|  | Engagement with civil society organizations, health authorities, and education networks |
|  | Development of communication strategies and materials for diverse target groups |
|  | Meetings with the Ministry of Health to align education and outreach efforts |
| 11-14 | **Phase 8: Integration of WGS into National Information Systems** |
|  | Technical integration of WGS results into the National Health Data Network (RNDS) and associated health information systems (e.g., GAL, Site-TB) |
| 6-23 | **Phase 9: GEMIBRA Platform Development** |
|  | Construction of a centralized mutation database |
|  | Development and deployment of a user-friendly interface with spatial analysis capability |
| 12-24 | **Phase 10: Impact Evaluation and Final Reporting** |
|  | Evaluation of WGS impact on diagnostic time, treatment decisions, and patient outcomes |
|  | Assessment of the effectiveness of training programs and community engagement |
|  | Production of interim and final technical reports |
